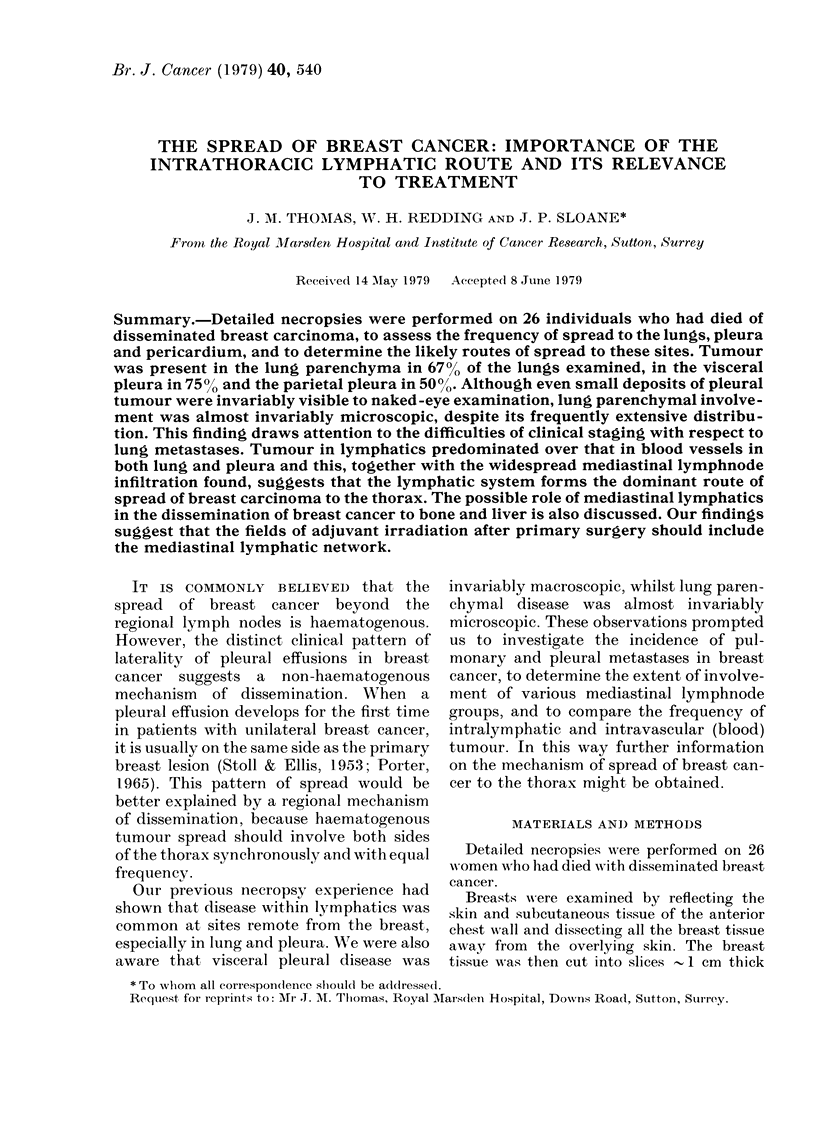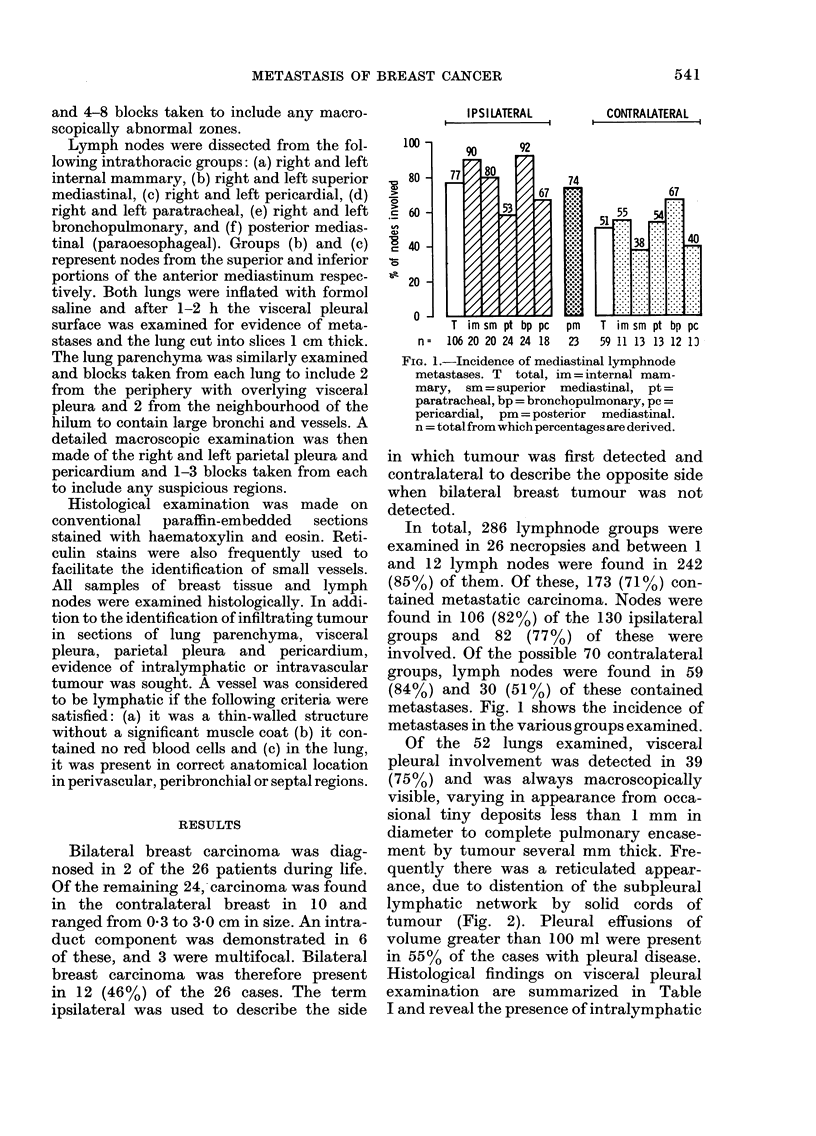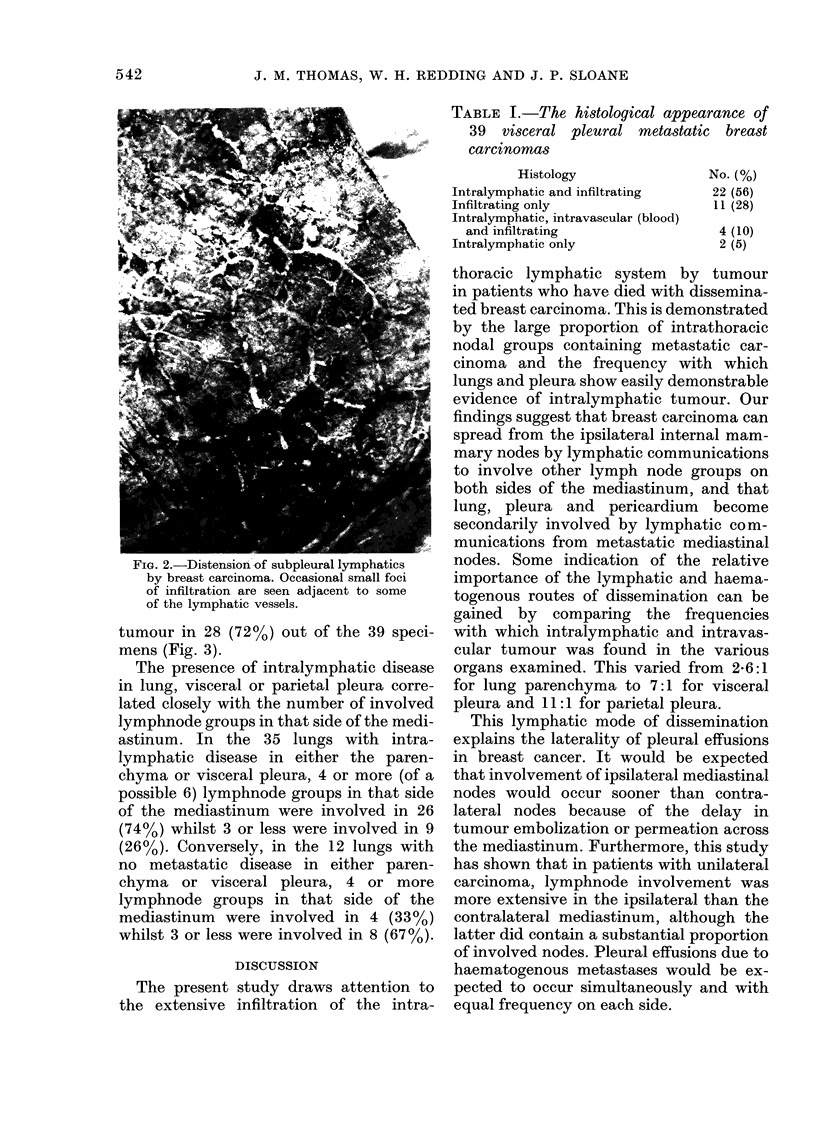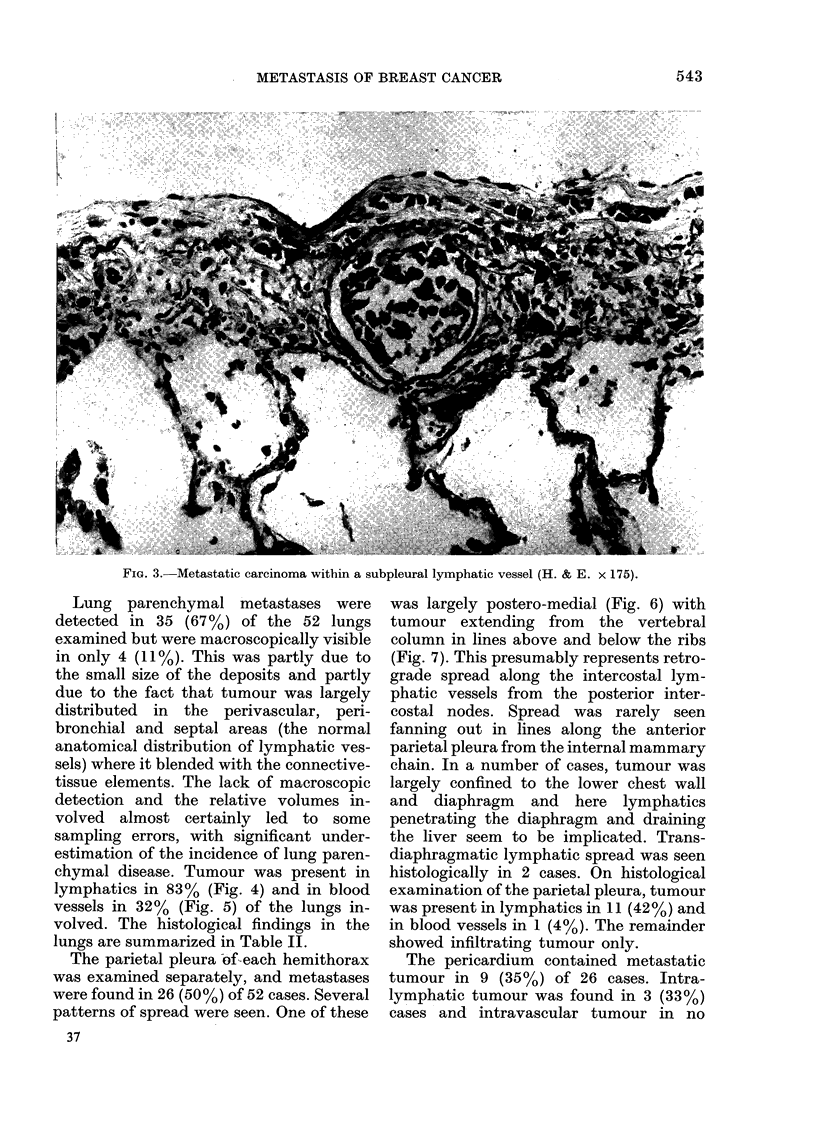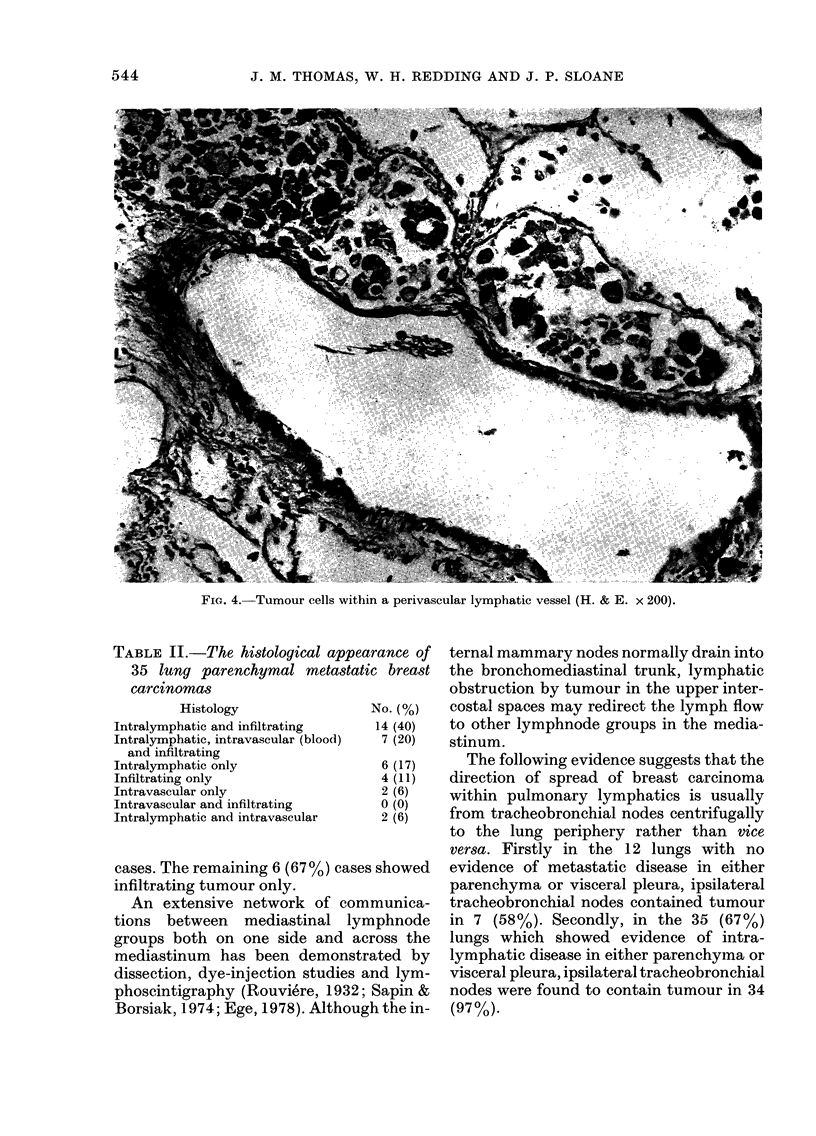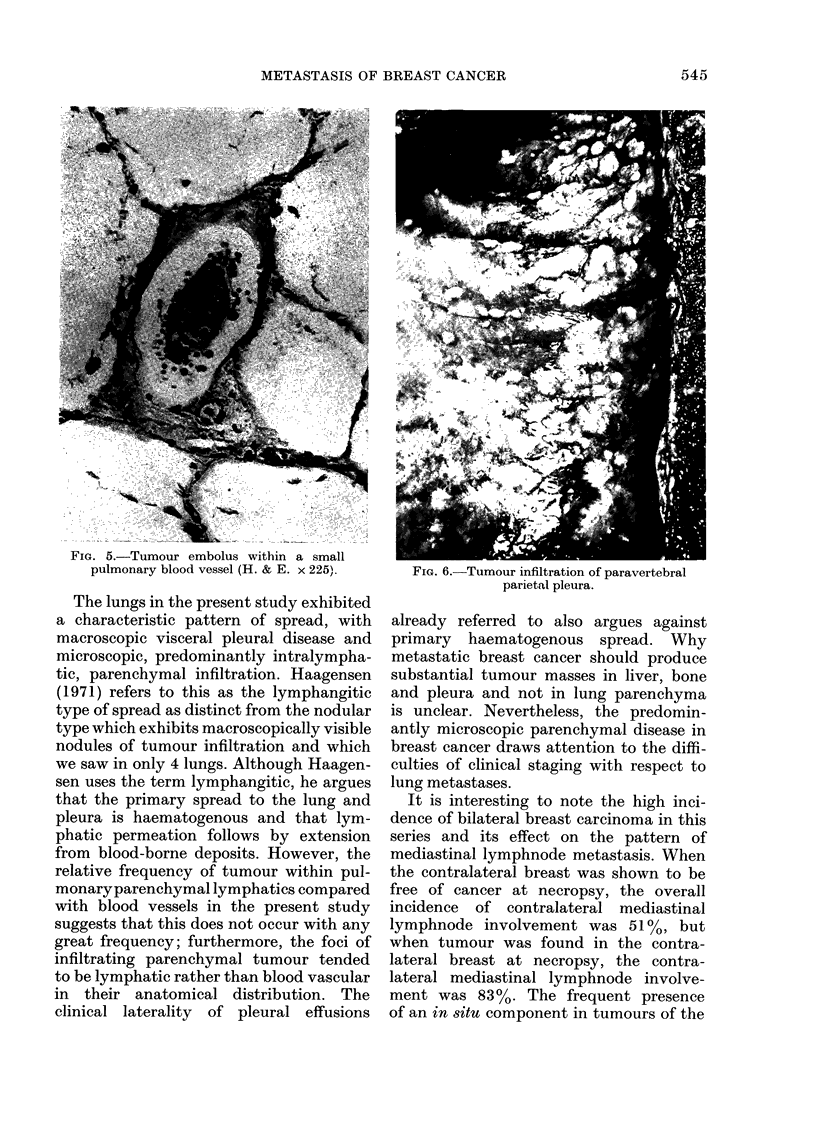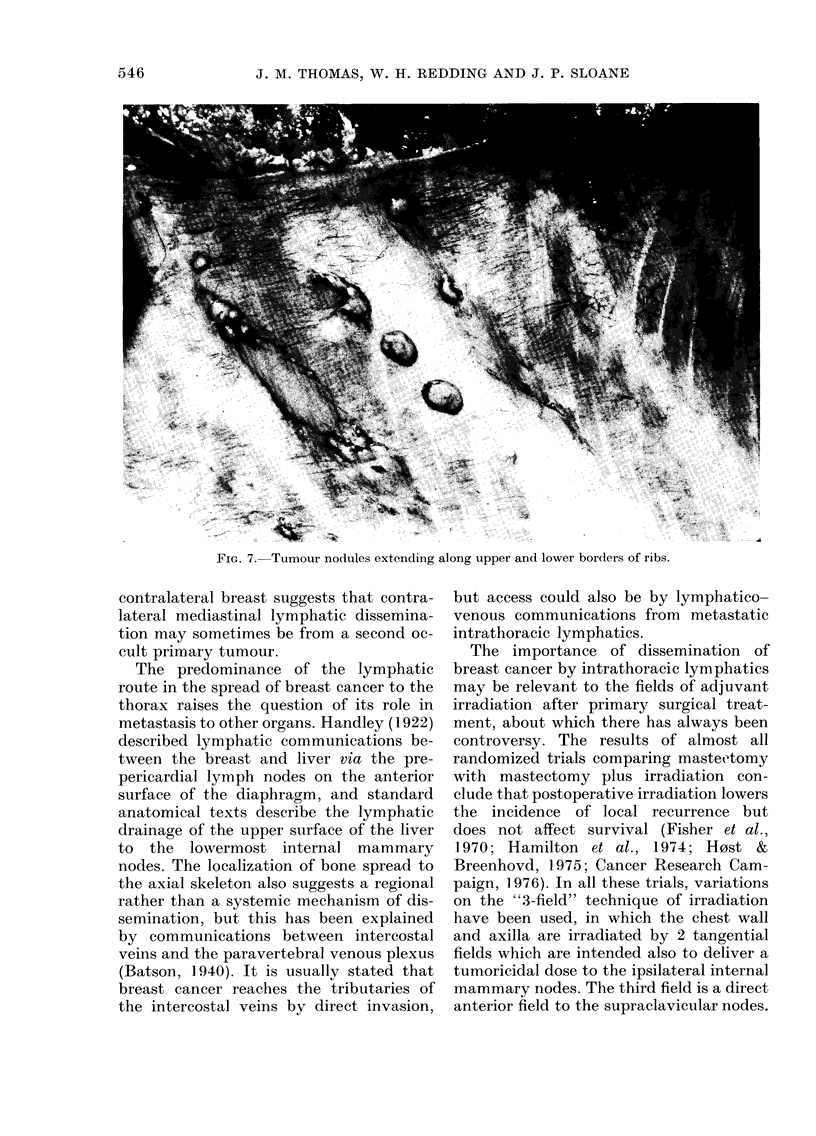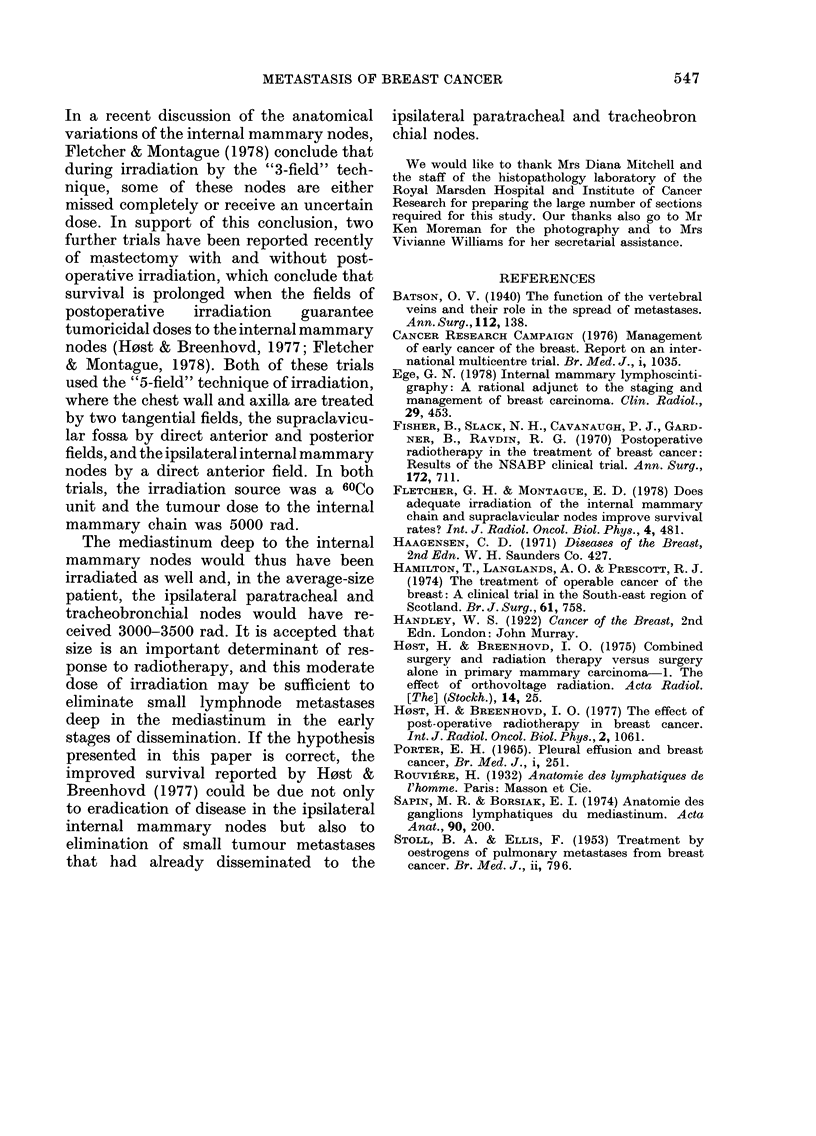# The spread of breast cancer: importance of the intrathoracic lymphatic route and its relevance to treatment.

**DOI:** 10.1038/bjc.1979.219

**Published:** 1979-10

**Authors:** J. M. Thomas, W. H. Redding, J. P. Sloane

## Abstract

**Images:**


*Br. J. Cancer* (1979) **40,** 540

### THE SPREAD OF BREAST CANCER: IMPORTANCE OF THE INTRATHORACIC LYMPHATIC ROUTE AND ITS RELEVANCE TO TREATMENT

J. M. THOMAS, W. H. REDDING AND J. P. SLOANE*

*From the Royal Marsden Hospital and Institute of Cancer Research, Sutton, Surrey*

Received 14 May 1979 Accepted 8 June 1979

**Summary.—Detailed necropsies were performed on 26 individuals who had died of disseminated breast carcinoma, to assess the frequency of spread to the lungs, pleura and pericardium, and to determine the likely routes of spread to these sites. Tumour was present in the lung parenchyma in 67% of the lungs examined, in the visceral pleura in 75% and the parietal pleura in 50%. Although even small deposits of pleural tumour were invariably visible to naked-eye examination, lung parenchymal involvement was almost invariably microscopic, despite its frequently extensive distribution. This finding draws attention to the difficulties of clinical staging with respect to lung metastases. Tumour in lymphatics predominated over that in blood vessels in both lung and pleura and this, together with the widespread mediastinal lymphnode infiltration found, suggests that the lymphatic system forms the dominant route of spread of breast carcinoma to the thorax. The possible role of mediastinal lymphatics in the dissemination of breast cancer to bone and liver is also discussed. Our findings suggest that the fields of adjuvant irradiation after primary surgery should include the mediastinal lymphatic network.**

IT IS COMMONLY BELIEVED that the spread of breast cancer beyond the regional lymph nodes is haematogenous. However, the distinct clinical pattern of laterality of pleural effusions in breast cancer suggests a non-haematogenous mechanism of dissemination. When a pleural effusion develops for the first time in patients with unilateral breast cancer, it is usually on the same side as the primary breast lesion (Stoll & Ellis, 1953; Porter, 1965). This pattern of spread would be better explained by a regional mechanism of dissemination, because haematogenous tumour spread should involve both sides of the thorax synchronously and with equal frequency.

Our previous necropsy experience had shown that disease within lymphatics was common at sites remote from the breast, especially in lung and pleura. We were also aware that visceral pleural disease was invariably macroscopic, whilst lung parenchymal disease was almost invariably microscopic. These observations prompted us to investigate the incidence of pulmonary and pleural metastases in breast cancer, to determine the extent of involvement of various mediastinal lymphnode groups, and to compare the frequency of intralymphatic and intravascular (blood) tumour. In this way further information on the mechanism of spread of breast cancer to the thorax might be obtained.

### MATERIALS AND METHODS

Detailed necropsies were performed on 26 women who had died with disseminated breast cancer.

Breasts were examined by reflecting the skin and subcutaneous tissue of the anterior chest wall and dissecting all the breast tissue away from the overlying skin. The breast tissue was then cut into slices ~1 cm thick

* To whom all correspondence should be addressed.

Request for reprints to: Mr J. M. Thomas, Royal Marsden Hospital, Downs Road, Sutton, Surrey.

and 4–8 blocks taken to include any macroscopically abnormal zones.

Lymph nodes were dissected from the following intrathoracic groups: (a) right and left internal mammary, (b) right and left superior mediastinal, (c) right and left pericardial, (d) right and left paratracheal, (e) right and left bronchopulmonary, and (f) posterior mediastinal (paraoesophageal). Groups (b) and (c) represent nodes from the superior and inferior portions of the anterior mediastinum respectively. Both lungs were inflated with formol saline and after 1–2 h the visceral pleural surface was examined for evidence of metastases and the lung cut into slices 1 cm thick. The lung parenchyma was similarly examined and blocks taken from each lung to include 2 from the periphery with overlying visceral pleura and 2 from the neighbourhood of the hilum to contain large bronchi and vessels. A detailed macroscopic examination was then made of the right and left parietal pleura and pericardium and 1–3 blocks taken from each to include any suspicious regions.

Histological examination was made on conventional paraffin-embedded sections stained with haematoxylin and eosin. Reticulin stains were also frequently used to facilitate the identification of small vessels. All samples of breast tissue and lymph nodes were examined histologically. In addition to the identification of infiltrating tumour in sections of lung parenchyma, visceral pleura, parietal pleura and pericardium, evidence of intralymphatic or intravascular tumour was sought. A vessel was considered to be lymphatic if the following criteria were satisfied: (a) it was a thin-walled structure without a significant muscle coat (b) it contained no red blood cells and (c) in the lung, it was present in correct anatomical location in perivascular, peribronchial or septal regions.

### RESULTS

Bilateral breast carcinoma was diagnosed in 2 of the 26 patients during life. Of the remaining 24, carcinoma was found in the contralateral breast in 10 and ranged from 0·3 to 3·0 cm in size. An intraduct component was demonstrated in 6 of these, and 3 were multifocal. Bilateral breast carcinoma was therefore present in 12 (46%) of the 26 cases. The term ipsilateral was used to describe the side

FIG. 1.—Incidence of mediastinal lymphnode metastases. T total, im = internal mammary, sm = superior mediastinal, pt = paratracheal, bp = bronchopulmonary, pc = pericardial, pm = posterior mediastinal. n = total from which percentages are derived.

in which tumour was first detected and contralateral to describe the opposite side when bilateral breast tumour was not detected.

In total, 286 lymphnode groups were examined in 26 necropsies and between 1 and 12 lymph nodes were found in 242 (85%) of them. Of these, 173 (71%) contained metastatic carcinoma. Nodes were found in 106 (82%) of the 130 ipsilateral groups and 82 (77%) of these were involved. Of the possible 70 contralateral groups, lymph nodes were found in 59 (84%) and 30 (51%) of these contained metastases. Fig. 1 shows the incidence of metastases in the various groups examined.

Of the 52 lungs examined, visceral pleural involvement was detected in 39 (75%) and was always macroscopically visible, varying in appearance from occasional tiny deposits less than 1 mm in diameter to complete pulmonary encasement by tumour several mm thick. Frequently there was a reticulated appearance, due to distention of the subpleural lymphatic network by solid cords of tumour (Fig. 2). Pleural effusions of volume greater than 100 ml were present in 55% of the cases with pleural disease. Histological findings on visceral pleural examination are summarized in Table I and reveal the presence of intralymphatic

FIG. 2.—Distension of subpleural lymphatics by breast carcinoma. Occasional small foci of infiltration are seen adjacent to some of the lymphatic vessels.

tumour in 28 (72%) out of the 39 specimens (Fig. 3).

The presence of intralymphatic disease in lung, visceral or parietal pleura correlated closely with the number of involved lymphnode groups in that side of the mediastinum. In the 35 lungs with intralymphatic disease in either the parenchyma or visceral pleura, 4 or more (of a possible 6) lymphnode groups in that side of the mediastinum were involved in 26 (74%) whilst 3 or less were involved in 9 (26%). Conversely, in the 12 lungs with no metastatic disease in either parenchyma or visceral pleura, 4 or more lymphnode groups in that side of the mediastinum were involved in 4 (33%) whilst 3 or less were involved in 8 (67%).

### DISCUSSION

The present study draws attention to the extensive infiltration of the intra-

TABLE I.—*The histological appearance of 39 visceral pleural metastatic breast carcinomas*

| Histology | No. (%) |
|---|---|
| Intralymphatic and infiltrating | 22 (56) |
| Infiltrating only | 11 (28) |
| Intralymphatic, intravascular (blood) and infiltrating | 4 (10) |
| Intralymphatic only | 2 (5) |

thoracic lymphatic system by tumour in patients who have died with disseminated breast carcinoma. This is demonstrated by the large proportion of intrathoracic nodal groups containing metastatic carcinoma and the frequency with which lungs and pleura show easily demonstrable evidence of intralymphatic tumour. Our findings suggest that breast carcinoma can spread from the ipsilateral internal mammary nodes by lymphatic communications to involve other lymph node groups on both sides of the mediastinum, and that lung, pleura and pericardium become secondarily involved by lymphatic communications from metastatic mediastinal nodes. Some indication of the relative importance of the lymphatic and haematogenous routes of dissemination can be gained by comparing the frequencies with which intralymphatic and intravascular tumour was found in the various organs examined. This varied from 2·6:1 for lung parenchyma to 7:1 for visceral pleura and 11:1 for parietal pleura.

This lymphatic mode of dissemination explains the laterality of pleural effusions in breast cancer. It would be expected that involvement of ipsilateral mediastinal nodes would occur sooner than contralateral nodes because of the delay in tumour embolization or permeation across the mediastinum. Furthermore, this study has shown that in patients with unilateral carcinoma, lymphnode involvement was more extensive in the ipsilateral than the contralateral mediastinum, although the latter did contain a substantial proportion of involved nodes. Pleural effusions due to haematogenous metastases would be expected to occur simultaneously and with equal frequency on each side.

FIG. 3.—Metastatic carcinoma within a subpleural lymphatic vessel (H. & E. ×175).

Lung parenchymal metastases were detected in 35 (67%) of the 52 lungs examined but were macroscopically visible in only 4 (11%). This was partly due to the small size of the deposits and partly due to the fact that tumour was largely distributed in the perivascular, peribronchial and septal areas (the normal anatomical distribution of lymphatic vessels) where it blended with the connective-tissue elements. The lack of macroscopic detection and the relative volumes involved almost certainly led to some sampling errors, with significant underestimation of the incidence of lung parenchymal disease. Tumour was present in lymphatics in 83% (Fig. 4) and in blood vessels in 32% (Fig. 5) of the lungs involved. The histological findings in the lungs are summarized in Table II.

The parietal pleura of each hemithorax was examined separately, and metastases were found in 26 (50%) of 52 cases. Several patterns of spread were seen. One of these was largely postero-medial (Fig. 6) with tumour extending from the vertebral column in lines above and below the ribs (Fig. 7). This presumably represents retrograde spread along the intercostal lymphatic vessels from the posterior intercostal nodes. Spread was rarely seen fanning out in lines along the anterior parietal pleura from the internal mammary chain. In a number of cases, tumour was largely confined to the lower chest wall and diaphragm and here lymphatics penetrating the diaphragm and draining the liver seem to be implicated. Trans-diaphragmatic lymphatic spread was seen histologically in 2 cases. On histological examination of the parietal pleura, tumour was present in lymphatics in 11 (42%) and in blood vessels in 1 (4%). The remainder showed infiltrating tumour only.

The pericardium contained metastatic tumour in 9 (35%) of 26 cases. Intra-lymphatic tumour was found in 3 (33%) cases and intravascular tumour in no

FIG. 4.—Tumour cells within a perivascular lymphatic vessel (H. & E. × 200).

TABLE II.—*The histological appearance of 35 lung parenchymal metastatic breast carcinomas*

| Histology | No. (%) |
|---|---|
| Intralymphatic and infiltrating | 14 (40) |
| Intralymphatic, intravascular (blood) and infiltrating | 7 (20) |
| Intralymphatic only | 6 (17) |
| Infiltrating only | 4 (11) |
| Intravascular only | 2 (6) |
| Intravascular and infiltrating | 0 (0) |
| Intralymphatic and intravascular | 2 (6) |

cases. The remaining 6 (67%) cases showed infiltrating tumour only.

An extensive network of communications between mediastinal lymphnode groups both on one side and across the mediastinum has been demonstrated by dissection, dye-injection studies and lymphoscintigraphy (Rouviére, 1932; Sapin & Borsiak, 1974; Ege, 1978). Although the internal mammary nodes normally drain into the bronchomediastinal trunk, lymphatic obstruction by tumour in the upper intercostal spaces may redirect the lymph flow to other lymphnode groups in the mediastinum.

The following evidence suggests that the direction of spread of breast carcinoma within pulmonary lymphatics is usually from tracheobronchial nodes centrifugally to the lung periphery rather than *vice versa*. Firstly in the 12 lungs with no evidence of metastatic disease in either parenchyma or visceral pleura, ipsilateral tracheobronchial nodes contained tumour in 7 (58%). Secondly, in the 35 (67%) lungs which showed evidence of intralymphatic disease in either parenchyma or visceral pleura, ipsilateral tracheobronchial nodes were found to contain tumour in 34 (97%).

FIG. 5.—Tumour embolus within a small pulmonary blood vessel (H. & E. × 225).

FIG. 6.—Tumour infiltration of paravertebral parietal pleura.

The lungs in the present study exhibited a characteristic pattern of spread, with macroscopic visceral pleural disease and microscopic, predominantly intralymphatic, parenchymal infiltration. Haagensen (1971) refers to this as the lymphangitic type of spread as distinct from the nodular type which exhibits macroscopically visible nodules of tumour infiltration and which we saw in only 4 lungs. Although Haagensen uses the term lymphangitic, he argues that the primary spread to the lung and pleura is haematogenous and that lymphatic permeation follows by extension from blood-borne deposits. However, the relative frequency of tumour within pulmonary parenchymal lymphatics compared with blood vessels in the present study suggests that this does not occur with any great frequency; furthermore, the foci of infiltrating parenchymal tumour tended to be lymphatic rather than blood vascular in their anatomical distribution. The clinical laterality of pleural effusions already referred to also argues against primary haematogenous spread. Why metastatic breast cancer should produce substantial tumour masses in liver, bone and pleura and not in lung parenchyma is unclear. Nevertheless, the predominantly microscopic parenchymal disease in breast cancer draws attention to the difficulties of clinical staging with respect to lung metastases.

It is interesting to note the high incidence of bilateral breast carcinoma in this series and its effect on the pattern of mediastinal lymphnode metastasis. When the contralateral breast was shown to be free of cancer at necropsy, the overall incidence of contralateral mediastinal lymphnode involvement was 51%, but when tumour was found in the contralateral breast at necropsy, the contralateral mediastinal lymphnode involvement was 83%. The frequent presence of an *in situ* component in tumours of the

FIG. 7.—Tumour nodules extending along upper and lower borders of ribs.

contralateral breast suggests that contralateral mediastinal lymphatic dissemination may sometimes be from a second occult primary tumour.

The predominance of the lymphatic route in the spread of breast cancer to the thorax raises the question of its role in metastasis to other organs. Handley (1922) described lymphatic communications between the breast and liver *via* the prepericardial lymph nodes on the anterior surface of the diaphragm, and standard anatomical texts describe the lymphatic drainage of the upper surface of the liver to the lowermost internal mammary nodes. The localization of bone spread to the axial skeleton also suggests a regional rather than a systemic mechanism of dissemination, but this has been explained by communications between intercostal veins and the paravertebral venous plexus (Batson, 1940). It is usually stated that breast cancer reaches the tributaries of the intercostal veins by direct invasion, but access could also be by lymphatico–venous communications from metastatic intrathoracic lymphatics.

The importance of dissemination of breast cancer by intrathoracic lymphatics may be relevant to the fields of adjuvant irradiation after primary surgical treatment, about which there has always been controversy. The results of almost all randomized trials comparing mastectomy with mastectomy plus irradiation conclude that postoperative irradiation lowers the incidence of local recurrence but does not affect survival (Fisher *et al.*, 1970; Hamilton *et al.*, 1974; Høst & Breenhovd, 1975; Cancer Research Campaign, 1976). In all these trials, variations on the "3-field" technique of irradiation have been used, in which the chest wall and axilla are irradiated by 2 tangential fields which are intended also to deliver a tumoricidal dose to the ipsilateral internal mammary nodes. The third field is a direct anterior field to the supraclavicular nodes.

In a recent discussion of the anatomical variations of the internal mammary nodes, Fletcher & Montague (1978) conclude that during irradiation by the "3-field" technique, some of these nodes are either missed completely or receive an uncertain dose. In support of this conclusion, two further trials have been reported recently of mastectomy with and without postoperative irradiation, which conclude that survival is prolonged when the fields of postoperative irradiation guarantee tumoricidal doses to the internal mammary nodes (Høst & Breenhovd, 1977; Fletcher & Montague, 1978). Both of these trials used the "5-field" technique of irradiation, where the chest wall and axilla are treated by two tangential fields, the supraclavicular fossa by direct anterior and posterior fields, and the ipsilateral internal mammary nodes by a direct anterior field. In both trials, the irradiation source was a $^{60}$Co unit and the tumour dose to the internal mammary chain was 5000 rad.

The mediastinum deep to the internal mammary nodes would thus have been irradiated as well and, in the average-size patient, the ipsilateral paratracheal and tracheobronchial nodes would have received 3000–3500 rad. It is accepted that size is an important determinant of response to radiotherapy, and this moderate dose of irradiation may be sufficient to eliminate small lymphnode metastases deep in the mediastinum in the early stages of dissemination. If the hypothesis presented in this paper is correct, the improved survival reported by Høst & Breenhovd (1977) could be due not only to eradication of disease in the ipsilateral internal mammary nodes but also to elimination of small tumour metastases that had already disseminated to the ipsilateral paratracheal and tracheobron chial nodes.

We would like to thank Mrs Diana Mitchell and the staff of the histopathology laboratory of the Royal Marsden Hospital and Institute of Cancer Research for preparing the large number of sections required for this study. Our thanks also go to Mr Ken Moreman for the photography and to Mrs Vivianne Williams for her secretarial assistance.

### REFERENCES

Batson, O. V. (1940) The function of the vertebral veins and their role in the spread of metastases. *Ann. Surg.*, **112**, 138.

Cancer Research Campaign (1976) Management of early cancer of the breast. Report on an international multicentre trial. *Br. Med. J.*, i, 1035.

Ege, G. N. (1978) Internal mammary lymphoscintigraphy: A rational adjunct to the staging and management of breast carcinoma. *Clin. Radiol.*, **29**, 453.

Fisher, B., Slack, N. H., Cavanaugh, P. J., Gardner, B., Ravdin, R. G. (1970) Postoperative radiotherapy in the treatment of breast cancer: Results of the NSABP clinical trial. *Ann. Surg.*, **172**, 711.

Fletcher, G. H. & Montague, E. D. (1978) Does adequate irradiation of the internal mammary chain and supraclavicular nodes improve survival rates? *Int. J. Radiol. Oncol. Biol. Phys.*, **4**, 481.

Haagensen, C. D. (1971) *Diseases of the Breast*, 2nd Edn. W. H. Saunders Co. 427.

Hamilton, T., Langlands, A. O. & Prescott, R. J. (1974) The treatment of operable cancer of the breast: A clinical trial in the South-east region of Scotland. *Br. J. Surg.*, **61**, 758.

Handley, W. S. (1922) *Cancer of the Breast*, 2nd Edn. London: John Murray.

Høst, H. & Breenhovd, I. O. (1975) Combined surgery and radiation therapy versus surgery alone in primary mammary carcinoma—1. The effect of orthovoltage radiation. *Acta Radiol.* [*The*] (*Stockh.*), **14**, 25.

Høst, H. & Breenhovd, I. O. (1977) The effect of post-operative radiotherapy in breast cancer. *Int. J. Radiol. Oncol. Biol. Phys.*, **2**, 1061.

Porter, E. H. (1965). Pleural effusion and breast cancer, *Br. Med. J.*, i, 251.

Rouviére, H. (1932) *Anatomie des lymphatiques de l'homme*. Paris: Masson et Cie.

Sapin, M. R. & Borsiak, E. I. (1974) Anatomie des ganglions lymphatiques du mediastinum. *Acta Anat.*, **90**, 200.

Stoll, B. A. & Ellis, F. (1953) Treatment by oestrogens of pulmonary metastases from breast cancer. *Br. Med. J.*, ii, 796.